# Study of Survival Rate After Cardiopulmonary Resuscitation (CPR) in Hospitals of Kermanshah in 2013

**DOI:** 10.5539/gjhs.v7n1p52

**Published:** 2014-07-30

**Authors:** Afshin Goodarzi, Amir Jalali, Afshin Almasi, Arsalan Naderipour, Reza Pourmirza Kalhori, Amineh Khodadadi

**Affiliations:** 1 Department of Emergency Medicine, School of Paramedics, Kermanshah University of Medical Sciences (KUMS), Kermanshah, Iran; 2 Psychiatric Nursing Department, Faculty of Nursing and Midwifery, Kermanshah University of Medical Sciences (KUMS), Kermanshah, Iran; 3 Department of Epidemiology and Biostatistics, Tehran University of Medical Sciences (TUMS), Tehran, Iran; 4 Bs Student in Midwifery, Kermanshah University of Medical Sciences (KUMS), Kermanshah, Iran

**Keywords:** cardio-pulmonary resuscitation (CPR), resuscitation success, follow-up

## Abstract

**Background:**

After CPR, the follow-up of survival rate and caused complications are the most important practices of the medical group. This study was performed aimed at determining the follow-up results after CPR in patients of university hospitals in Kermanshah in 2014.

**Methods:**

In this prospective study, 320 samples were examined. A purposive sampling method was used, and data was collected using a researcher-made information form with content and face validity and reliability of r= 0.79. Data was analyzed with STATA9 software and statistical tests, including calculation of the success rate, relative risk (RR), chi-square and Fisher at significance level of P < 0.05.

**Results:**

The initial success rate of cardiopulmonary resuscitation was equal to 15.3%, while the ultimate success rate (discharged alive from the hospital) was as 10.6%. The six-month success rate after resuscitation was 8.78% than those who were discharged alive. There were no significant statistical differences between different age groups regarding the initial success rate of resuscitation (P = 0.14), and the initial resuscitation success rate was higher in patients in morning shift (P = 0.02).

**Conclusion:**

By the results of study, it is recommended to increase the medical - nursing knowledge and techniques for personnel in the evening and night shifts. Also, an appropriate dissemination of health care staff in working shifts should be done to increase the success rate of CPR procedure.

## 1. Introduction

Cardiac arrest means the stop of heart rates, which is followed by stopping the blood supply to the brain and other vital organs. Studies show that 400,000 people per year in America (Hazinski, 2011, Cave et al., 2010) and 700,000 people per year in Europe develop sudden cardiac arrest (Sans et al., 1997, Travers et al., 2010). Cardiac arrest kills 250,000 people in the U.S. annually (Loscalzo, 2010).

Several studies indicate that 70-85% of cases of cardiac arrest occur due to ventricular fibrillation. Other causes include ischemic heart diseases, valvular diseases, cardiac tamponed, pulmonary thromboembolism, aortic aneurysm, drug poisoning and overdoses of opioids and drugs (Irwin et al., 2008; Bryan et al., 2012). Following cardiac arrest, doing CPR as soon as possible after can lead to the patient resuscitation (Cave et al., 2010). Cardiopulmonary resuscitation procedure includes measures that are performed at the time of heart and lung arrest to restore the function of pulmonary ventilation and heart rate to prevent brain death (Salari et al., 2010).

Studies show that 14.3% of patients experiencing cardiac arrest are undergone CPR before transport to the hospital, and almost all of them will undergo cardiopulmonary resuscitation at the hospital (Loscalzo, 2010). In recent years, new major developments have been made in CPR operations using drugs, electroshock, and advanced measures of cardiac resuscitation (Marini & Wheeler, 2010). However, despite the great advances in treatment, the prognosis of resuscitation procedure is still poor (Rea et al., 2003; DeVreede-Swagemakers et al., 1997; De-Vos et al., 1999).

The primary measure of success of CPR is returning the heartbeat, which is determined by cardiac monitoring and central and peripheral pulse touching, and the ultimate measure of success is discharged alive from the hospital (Jafarian, 2002). In England (1999), from 828 patients undergoing CPR, 162 patients (20%) survived to discharge time, among which 5 cases developed vegetative state, and 51 cases died during the first few days after discharge (De-Vos et al., 1999). According to the results of domestic studies, the short-term survival rate was calculated as 32% (Nasiripour et al., 2012) in emergency rooms of Social Security in Alborz Province during 2012; in Shohadaye Haftom Tir hospital in Tehran (2001), this rate at initial success and ultimate success was reported as 29.3% and 10%, respectively (Jafarian, 2002). Also, in another study in the selected educational hospitals in Tehran (2007), the initial success rate was equal to 28.4%, while the final success rate was as 7.2% (Salari et al., 2010). Thomas et al. (1991) conducted a study in the UK on 948 CPR patients adults aged over 18 years. The results showed that 298 (32.2%) patients were discharged alive from the hospital that 24.5% of them died within 1 year (Thomas et al., 2000). In U.S (1992), 24 patients (29%) of 83 patients given CPR were discharged from the hospital alive that 13 patients (54%) of discharged ones survived up to 31 months after discharge. The results of this study show a significant relationship between cardiac dysrhythmia and patients’ survival after cardiac resuscitation procedure. There is also a significant relationship between the duration of cardiopulmonary resuscitation performing (less or more than 10 minutes) and patients’ survival (Robinson, 1994). The results of studies in other countries have reported the initial success rate of resuscitation up to 61.2% at maximum and ultimate success rate (discharge) up to 32.2% (Thomas et al., 2000). The rate in Iran was reported as 15.2% (Saifi et al., 2010).

Today, the available information in Iran’s hospitals is related to the initial success rate of CPR, and fewer studies have been conducted on final success rate (discharge alive from hospital after initial success in CPR) and long-term follow-ups after discharge. For this reason, most therapist groups think of the result operation with disbelief. Thus, the possibility of reduced effort and seriousness to perform required measures at this critical moment is not unexpected. The purpose of this study is to determine the survival rate after cardiopulmonary resuscitation in educational-university centers in the city of Kermanshah in 2013-2014.

## 2. Materials and Methods

In this prospective study, the research environment included university educational health centers in the city of Kermanshah. Referring to these centers and considering the circumstances, the samples were selected based on objective and sequentially to participate in the study. Thus, the 99-code forms were obtained daily from the hospital nursing office. Then, referring to the relevant ward, the patient was followed up through his/her medical file. In case of patient death immediately after initial resuscitation, only the demographic information and the first part of the worksheet data collection were completed using the patient records. In case of initial success of resuscitation (heartbeat return), in addition to completing the first part of the data collection form, the patient was followed up by a research fellow at the hospital. If the patient was discharged alive, the second part of data collection form was completed, and the patient was monitored monthly by telephone for six months. At the period time, in case of patient death (due to earlier underlying problems and not because of the accidents and injuries) or due to lack of access, the patient was excluded from the study. It should be noted that in case of patient death in any stage before discharge or after the initial return of heartbeat, he was recorded as a patient with initial successful resuscitation.

The study population included all patients referred or admitted in university educational centers in the city of Kermanshah that CPR had been carried out for them as witnessed cardiac arrest or non-witnessed cardiac arrest. Inclusion criteria for this study included all patients, except for the followings: patients with duration time more than 6 minutes between cardiac arrest to beginning of CPR; patients with chronological age less than 12 years in children or more than 69 years on the elderly; patients with chronic failure in more than one of the vital organs, patients with metastatic malignancies and septic shock; patients that CPR are not recommended for them (patients without half of the head or body, full cutting of head and crushed brain) (Hazinski, 2011). According the values of α = 0.05, and the initial success rate equal to 0.281 (Saifi et al., 2010) and d = 0.05, the minimum sample size required was estimated by GPower software as 320 patients undergoing cardiopulmonary resuscitation operation, which was collected using the purposeful (object-oriented) sampling method.

Tools for data collection included a researcher-made data collection form that its validity was confirmed based on content measurement (using the comments of ten faculty members of Kermanshah University of Medical Sciences). Its reliability was also confirmed using a pilot study and with completing data collection sheets based on the files documents of 30 patients undergoing resuscitation by two expert advisors in the research and according to Kappa concordance coefficient (0.79). Data was analyzed by STATA9 software and through calculating the success rate and the relative risk (RR). For relationship assessment between other variables and survival rate of resuscitation (no death), the Chi-square test, the Chi-square test details, the Chi-square test for trend and Fisher’s exact test were used. In this study, the values of P < 0.05 were considered significant.

## 3. Results

The results showed that 4.68% of the studied samples were in the age range of 12–19 years, 42.5% in the age range of 20–39 years and 52.81% in the age range of 40–69 years. The sample included 57.5% male and 42.5% female. The most frequency of subjects according to the hospital admission ward was related to the emergency ward (72.18%) and the lowest was related to the CCU (1.25%). The study results showed that the initial success rate of cardiopulmonary resuscitation in the educational health centers in the city of Kermanshah was equal to 15.3% (49 patients) that the heartbeat return was evident in 9.1% (29 patients) of the samples during resuscitation, and in 6.2 % (20 cases), the heartbeat and respiration return were observed.

**Diagram 1:**
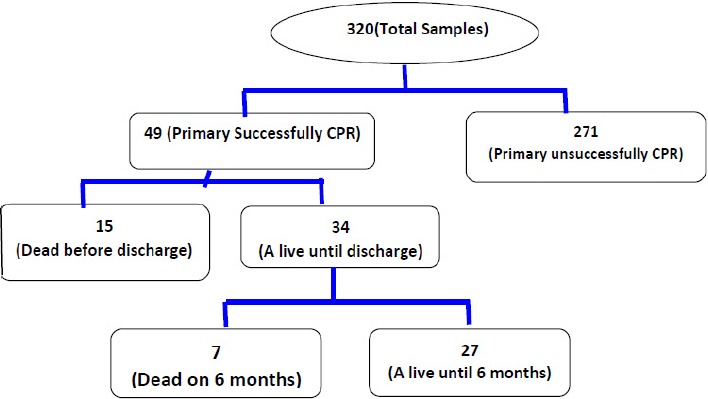
CPR outcomes of Samples study

The ultimate success rate of resuscitation (discharged alive from the hospital) was calculated as 10.6% (34 people), and at six-month follow-up survey data on patients after discharge, the long-term success rate of the resuscitation was as 78.8% (26 patients) than to the discharged and 8.12% of all the CPR-done patients. It should be noted that the conditions were not provided for performing the chi-square test to assess the association between the underlying disease and the success rate of CPR (Diagram 1).

No statistically significant relationship was observed between the gender and initial success rate (p = 031) and final success rate (P = 0.1) of resuscitation, despite the higher mortality risk in men as 1.04 times than women (RR = 1.04). Table 1 shows the initial and final success rates of resuscitation based on age. Although in the first age group, the risk of death was obtained as 1.18 and 1.17 times more than the second and third age groups, respectively, however, based on the Chi-square test for trend results, there was no statistically significant difference between the initial resuscitation success rates for different age groups (P-value = 0.14). Failing to establish the Chi-square test conditions, measurement the association between age and the ultimate success and long lasting CPR was not possible. Based on the results, the highest and lowest rates of initial success were related to the poisoning cases (33.3%) and malignancies (0%), respectively. Also, the success rate of cardiopulmonary resuscitation in traumatic patients was calculated as 12% (Table 1).

**Table 1:** Absolute and relative frequency of CPR outcome based underlying variables

Parameter	CPR outcome	Primary		Finally	

		Successful N (%)	Unsuccessful N (%)	Successful N (%)	Unsuccessful N (%)
Age	12-19	1(6.66)	14(93.33)	1(100)	0(0)
	20-39	24(17.64)	112(82.35)	19(79.2)	5(20.8)
	40-69	24(14.2)	145(85.5)	14(58.3)	10(41.7)
Working Shift	Morning	19(27.5)	50(72.5)	14(73.7)	5(26.3)
	Evening	20(15.3)	111(84.7)	15(75)	5(25)
	Night	10(8.3)	110(91.7)	5(50)	5(50)
Underlying disease	Internal	8(10.6)	67(89.4)	2(25)	6(75)
	Neurology	10(11.9)	74(88.1)	6(60)	4(40)
	Malignity	0(0)	10(100)	-	-
	Poisoning	10(33.33)	20(66.67)	10(100)	0(0)
	Trauma	4(12.1)	29(87.9)	3(75)	1(25)
	Surgical	5(15.6)	27(84.4)	5(100)	0(0)
	Cardiovascular	8(25)	24(75)	6(75)	2(25)
	Neurosurgery	4(12.5)	28(87.5)	2(50)	2(50)

The initial success rate of resuscitation had a significant relationship with working shifts (morning, afternoon, evening) (P-value = 0.02). Thus, according to the Chi-square test details the test results, the success rate in morning shift was higher than evening and night shifts (P-value = 0.018), and in evening shift more than the night shift (P-value = 0.04). While based on the Chi-square test, there was no significant relationship between the final success rate of resuscitation (discharged alive ones) and working shifts (P-value = 0.32) (Table 1).

## 4. Discussion

No statistically significant relationship was observed between the patients’ age and sex and CPR success rate. These results are consistent with the study conducted in 2012 in the hospitals of Alborz province (Nasiripour et al., 2012) and the conducted study at Imam Khomeini Hospital, Babol in 2010 (Brimnejad, 2009). The results show the initial success rate of resuscitation as 15.3%, which is lower than results reported in other studies as follows: 28.1% in Kermanshah (Saifi et al., 2010), 29.3% in Shohadaye Haftom Tir, Tehran (Jafarian, 2002), 28.4% in selected educational centers of Tehran (Salari et al., 2010), 19.9% in educational centers of Kashan (Adib-Hajbaghery et al., 2005), 32% in Social Security emergency hospitals of Alborz Province (Nasiripour et al., 2012), 39.7% in Brazil (Moretti et al., 2007) and in some countries such as the UK up to 61.2% (Thomas et al., 2000). In this regard, Philip (2012) states that despite the progress in medical procedures and medical devices, the results and prognosis in patients with sudden cardiopulmonary arrest is still weak (Philip & Jie, 2012).

The ultimate success rate in this study was equal to 10.6%, which was consistent with the results of studies conducted in 2001in Tehran (Jafarian, 2002)with a final success rate of 10%. It had a better status than the results of a study conducted in Kashan in 2003 with a rate of 5.3% (Adib-Hajbaghery et al., 2005) and the results of research done in selected educational centers of Tehran in 2007 with the success rate of 7.2 % (Salari et al., 2010). However, the alive discharged rates in this research was inconsistent with the results of the study conducted in Kermanshah in 2011 with a discharge rate of 15.7% (Saifi et al., 2010) and studies done in the UK in 1991with the ultimate success rate of 32.2% (Thomas et al., 2000) and in 1999 with the discharge rate of 20% (De-Vos et al., 1999), and the results of some studies that have mentioned the survival to discharge rates as 15% to 33% (Philip & Jie, 2012; Bellomo et al., 2003; Peberdy et al., 2003; Sandroni et al., 2004; Cooper & Evans, 2003).

The study results show that long-term success rate (survival after six months follow-up) of patients has been as 8.12% and 78.8% than to the total cases of CPR and to discharged alive ones, respectively. No similar study has been done on in Iran regarding this finding, but in a study conducted in Brazil (Moretti et al., 2007) on 156 patients during 2001, the one-year survival rate than to the patients undergone CPR was as 21.9%, which is higher than this study. In some cases, the weaker results have been reported, such as a research conducted at Atlanta Medical Center during 1995–2004 on 732 patients with the three-year survival rate of 41% than to the discharged ones (Bloom et al., 2007). In other countries, the rates compared to the total cases of CPR and to the discharged ones have been reported as follows that are consistent with the results of this study in some cases: 18% and 83% in Jiresaty Studies (1969) (Sans et al., 1997); 4% and 42% in Peatfield study (1977) (Peatfield et al., 1977); 11 % and 80% in Bedell et al. study (1983) (Bedell et al., 1983); 10% and 72% in Pechtel study (1984) (Suljaga-Pechtel et al., 1984); 12% and 72% in Pedo study (1992) (Tunstall-Pedoe et al., 1992).

Based on the study results, the greatest initial success rate of cardiopulmonary resuscitation was related to the poisoning cases (33.3%), and then the heart diseases (25%), while the lowest rate was related to the malignancies (0%), which are consistent with the results of a research conducted at the University Hospitals of Kashan in 2003 and overseas studies(Adib-Hajbaghery et al., 2005; Jiresaty et al., 1973; Wildsmith et al., 1972). The success rate of cardiopulmonary resuscitation in trauma patients was equal to 12%. These results are lower compared with the rates reported in other studies with success rate of 16% (Rea et al., 2003). Also, the success rate of resuscitation in patients with internal disease was 10.6%, which is lower compared to the results of a study conducted in 2001 in Tehran with a value of 14.6% (Jafarian, 2002).

The findings of a study conducted at the educational centers of Kashan in 2003 show that the highest and lowest success rates were related to the morning and evening shifts, respectively (Adib-Hajbaghery et al., 2005). In this study, the highest initial success rates of resuscitation were related in order to the morning, evening and night shifts, which are quite similar to the study results of the study conducted in Training centers in Tehran in 2007 (Salari et al., 2010). The low success rate of CPR can be due to the low number personnel and their lower readiness at night shift (Joseph, 2003). In this regard, Verhaegen and Khaleque in 1981 asserted that time differences and the circadian effects are only seen in shift workers under heavy pressure (Verhaegen et al., 1982; Travers et al., 2010).

## 5. Conclusion

In general, the results indicate the low immediate success rate (primary) of CPR, especially at night shifts. It seems that measures such as the following can be considered in planning to reduce the unsuccessful CPRs: Improving the skill level of the medical team; Formation an experienced CPR team; Retraining and up to dating medical and nursing knowledge and techniques; Formation of hospital resuscitation committees to review the existing problems in performed CPRs; Appropriate distribution of therapeutic staff on all three working shifts in order to mitigate the circadian effects; Daily calibration of equipment used in CPR; Review and standardization the physical space of CPR room.
